# ATP13A2-Mediated Spermine Export Modulates Lipid Catabolism in the Endolysosomal System of SH-SY5Y Cells

**DOI:** 10.3390/ijms27010484

**Published:** 2026-01-02

**Authors:** Alejandra L. Marcos, Mariela M. Gironacci, Felicitas de Tezanos Pinto

**Affiliations:** 1 Departamento de Química Biológica, Facultad de Farmacia y Bioquímica, Universidad de Buenos Aires (UBA), Junín 956, Buenos Aires 1113, Argentinamariela@qb.ffyb.uba.ar (M.M.G.); 2 Instituto de Química y Físicoquímica Biológicas, Consejo Nacional de Investigaciones Científicas y Tecnológicas, UBA-CONICET, Junín 956, Buenos Aires 1113, Argentina; 3 Instituto de Ciencias de la Salud, Universidad Nacional Arturo Jauretche, Av. Calchaquí, Buenos Aires 6200, Argentina

**Keywords:** P5B–ATP13A2-spermine export, ABHD6, GCase, ACase, endolysosomal lipid catabolism, neuronal ceroid lipofuscinosis, Parkinson’s disease

## Abstract

Dysfunction of the membrane transporter P5B-ATPase 13A2 (ATP13A2) has been linked to neurodegenerative disorders, while its overexpression has been associated with colorectal cancer. ATP13A2 localizes to lysosomes and late endosomes, where it exports polyamines such as spermine into the cytosol. We previously showed that ATP13A2 expression alters lipid homeostasis and reduces the levels of bis(monoacylglycero)phosphate (BMP), an anionic phospholipid essential for lipid digestion in acidic compartments, suggesting that ATP13A2-mediated spermine export may affect lysosomal lipid catabolism. α/β-hydrolase domain-containing 6 (ABHD6), the enzyme responsible for BMP catabolism, was detected by immunofluorescence and immunoblot analysis in SH-SY5Y cells overexpressing human ATP13A2 and treated with spermine. The activities of the lipid-degrading hydrolases acid ceramidase (ACase) and glucocerebrosidase (GCase) were measured using specific fluorogenic substrates. ATP13A2-expressing cells showed higher ABHD6 expression, and spermine treatment promoted its translocation to the cytoplasm. Spermine induced a transient increase in ACase activity, followed by a stronger inhibition in ATP13A2-expressing cells. Moreover, GCase activity was elevated in these cells but also showed greater spermine-induced inhibition. Altogether, these results suggest that ATP13A2-mediated spermine export modulates the lipid digestion capacity of the endolysosomal system and support a functional interplay between polyamine and lipid metabolism in these organelles.

## 1. Introduction

P-type ATPases belong to a superfamily of proteins that transport inorganic cations and other substrates across cell membranes through ATP hydrolysis. Based on sequence similarity they have been divided into five subfamilies termed P1-P5 [1]. The P5-ATPase subfamily is expressed only in eukaryotes and is divided into two groups termed P5A and P5B [2]. P5A-ATPases are found in the endoplasmic reticulum and influence protein maturation and secretion, whereas P5B-ATPases localized in late endosomes (also known as multivesicular bodies, MVBs) and lysosomes and are involved in the proper functioning of the endolysosomal system [3].

Loss-of-function mutations of the human gene coding for P5B-ATPase 13A2 (ATP13A2) underlie an early onset form of Parkinson’s called Kufor Rakeb Syndrome [4], as well as a type of Neuronal Ceroid Lipofusinosis [5], Hereditary Spastic Paraplegia [6] and Amyotrophic Lateral Sclerosis [7]. Moreover, upregulation of ATP13A2 activates the pentose phosphate pathway to promote colorectal cancer growth [8]. These observations suggest that the lack of a functional P5B-ATPase is primarily related to neuropathologies while its overexpression is involved in cancer development.

The substrate transported by P5B-ATPases was unknown until 2020, when Vangheluwe’s group demonstrated that P5B-ATPases export polyamines such as spermine from inside MVBs and lysosomes into the cytosol [9]. Subsequent studies using cryo-electron microscopy confirmed this finding [10,11,12,13,14,15]. In 2023, it was proposed that this enzyme might also act as a lysosomal H^+^/K^+^ transporter, resembling the mechanism of the gastric H^+^/K^+^ pump [16]. In addition, a preliminary study suggests that it may also act as a sphingomyelin-flippase, transferring this lipid from the inner to the outer leaflet of the endolysosomal membrane [17]. These results show that although the ATP dependent transport of polyamines is well established, there are still some controversial aspects that require clarification.

The constitutive degradation of membrane components inside intraluminal vesicles (ILVs) of MVBs requires a redistribution process in which cholesterol is exported from these ILVs while the levels of the anionic lipid bis(monoacylglycero)phosphate (BMP) increase [18,19]. This process enables certain acid hydrolases such as acid ceramidase (ACase), acid glucocerebrosidase (GCase) and acid sphingomyelinase—which are polycationic at the low pH 5 of these organelles—to bind to negatively charged ILVs due to the presence of BMP [20]. Once bound, these hydrolases are shielded from proteolytic breakdown, allowing them to efficiently act on their substrates. α/β Hydrolase domain-containing 6 (ABHD6) is the monoacylglycerol hydrolase responsible for BMP degradation. ABHD6 localizes in late endosomes and lysosomes, as well as other membrane compartments. However, since it is active at cytosolic pH and lacks acid hydrolase activity, it is thought to degrade BMP only after its export from ILVs to the endolysosomal limiting membrane [21]. BMP resides in MVBs and lysosomes, and is required not only for lipid degradation but also for exosome biogenesis within these acidic compartments [18,19,22]. ATP13A2 has emerged as a key regulator of endolysosomal function, since its dysfunction diminishes autophagic flux [20,21] and exosome release [23,24]. In line with this, we previously demonstrated that ATP13A2 expression alters lipid homeostasis in SH-SY5Y human neuroblastoma cells in a manner that favors lysosomal/exosomal secretion [25]. Moreover, we found that the expression of ATP13A2 reduces BMP content in these cells [25]. These results indicate that ATP13A2 may influence the lipid digestion capacity and/or lipid redistribution in these organelles. In this study, we tested the hypothesis that ATP13A2-driven spermine export modifies the enzymatic activities of ACase and GCase—two acid hydrolases involved in endolysosomal lipid catabolism—as well as the subcellular distribution of ABHD6. We found that ATP13A2 expression increased ABHD6 levels and spermine addition promoted its translocation to the cytoplasm. Furthermore, ACase and GCase activities were modified by spermine export driven by ATP13A2. Altogether, these findings suggest that ATP13A2-mediated spermine export may modulate the lipid digestion capacity of the endolysosomal system, supporting an interconnection between polyamine and lipid metabolism within these organelles.

## 2. Results

### 2.1. ABHD6 Expression Is Increased in ATP13A2-Expressing Cells and Spermine Treatment Favors Its Translocation Toward the Cytoplasm

**Figure 1:**
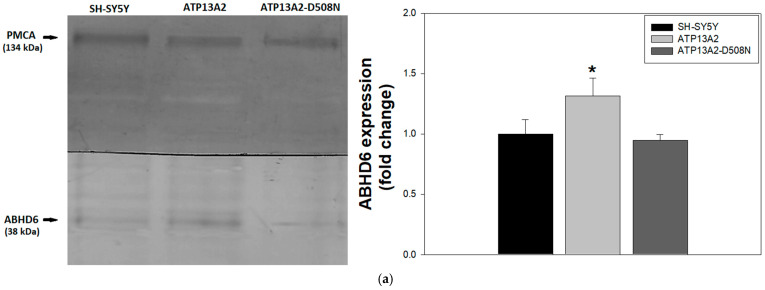

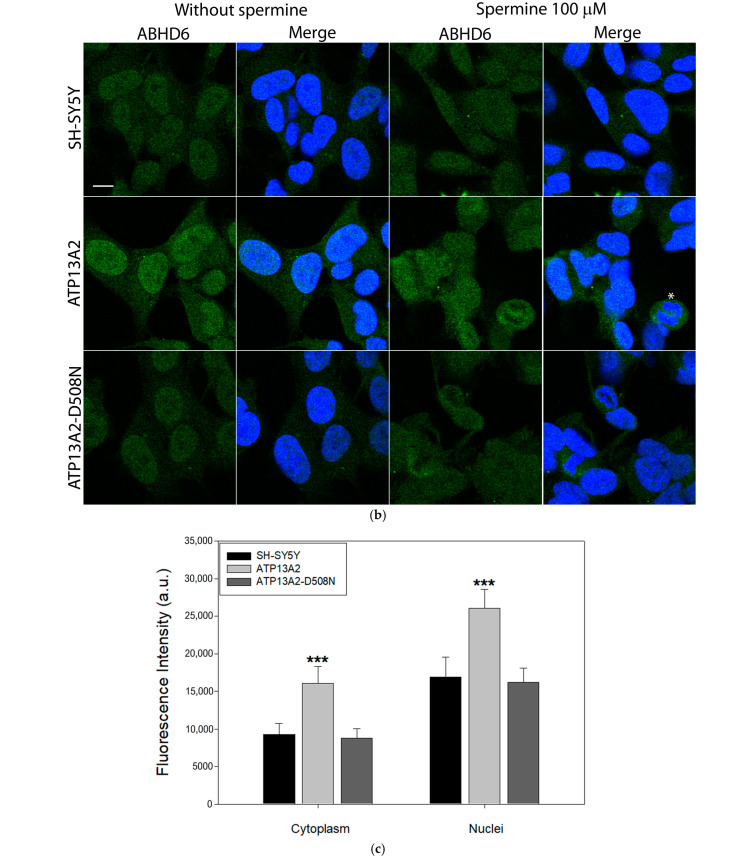

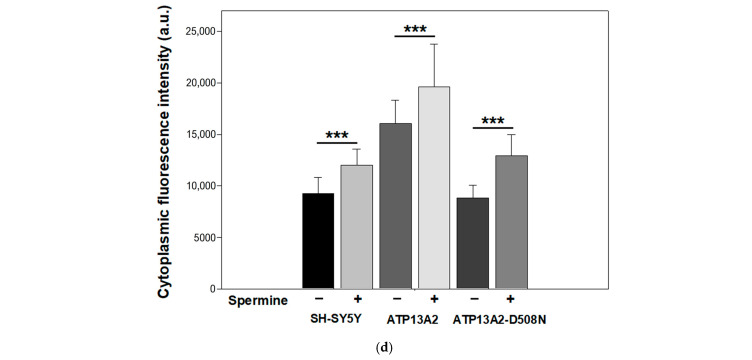
P5B-ATPase 13A2 (ATP13A2) expression increases α/β-hydrolase domain-containing 6 (ABHD6) levels and spermine promotes ABHD6 redistribution to the cytosol. (**a**) Ten micrograms of protein from homogenates of each cell line were separated by SDS-PAGE and transferred to PVDF membranes. ABHD6 was detected using a specific primary antibody (38 kDa band). Plasma membrane Ca^2+^-ATPase (PMCA) was used as a loading control (134 kDa band). The right panel shows densitometric quantification of ABHD6 normalized to PMCA for each sample. Data are expressed as fold change relative to SH-SY5Y cells and represent the mean ± SD of three independent experiments. * *p* < 0.05 vs. ATP13A2 cells; no significant differences were observed between SH-SY5Y and ATP13A2-D508N cells (ANOVA followed by Newman–Keuls multiple comparisons test). (**b**–**d**) Cells were incubated with 2.5 mM spermine at 37 °C for 2 h, fixed, permeabilized, and immunostained for ABHD6 as described in Section 4. Representative images show well-defined nuclear contours in untreated cells and reduced nuclear definition after spermine treatment. Nuclei were counterstained with DAPI. The asterisk indicates highly condensed chromatin corresponding to mitotic cells. Images were acquired using confocal fluorescence microscopy. Scale bar: 10 µm. Quantitative analysis of ABHD6 green fluorescence intensity is shown in (**c**,**d**). Panel (**c**) shows cytoplasmic and nuclear fluorescence in untreated cells, whereas panel (**d**) shows cytoplasmic fluorescence in cells treated or untreated with 2.5 mM spermine. Fluorescence intensity is expressed in arbitrary units (a.u.) and was quantified using ImageJ software (version 1.54k; National Institutes of Health, USA) by analyzing ≥50 cells from eight micrographs per cell line in three independent experiments. *** *p* < 0.001 vs. ATP13A2 cells (**c**) or vs. untreated cells (**d**) (ANOVA followed by Newman–Keuls multiple comparisons test).

As mentioned above, BMP plays a crucial role not only in facilitating lipid digestion within MVBs and lysosomes but also in promoting cholesterol efflux from ILVs and creating microdomains required for exosome biogenesis [22,26]. The decrease in BMP content observed in ATP13A2-expressing cells [25] suggests an alteration in BMP metabolism. To further investigate this possibility, we analyzed the content and subcellular distribution of ABHD6, the cytosolic enzyme responsible for BMP degradation [21]. Immunoblot analysis revealed increased ABHD6 expression in ATP13A2-expressing cells compared with controls (Figure 1a). Consistently, immunofluorescence analysis showed that SH-SY5Y and ATP13A2-D508N-expressing cells displayed similar ABHD6 fluorescence levels, whereas catalytically active ATP13A2 markedly increased both cytoplasmic and nuclear fluorescence intensity (Figure 1b, left panels; Figure 1c).

Based on the cell viability assays (Supplementary Figure S1), we selected 2.5 mM spermine as the highest concentration that did not compromise cell viability under our experimental conditions. At this concentration, spermine treatment promoted ABHD6 translocation toward the cytoplasm. Following spermine exposure, nuclear boundaries appeared less defined compared with untreated cells, in which nuclei were clearly delineated (Figure 1b, right panels). Quantitative image analysis confirmed a spermine-induced increase in cytoplasmic ABHD6 fluorescence across all cell lines evaluated (Figure 1d).

**Figure 2:**
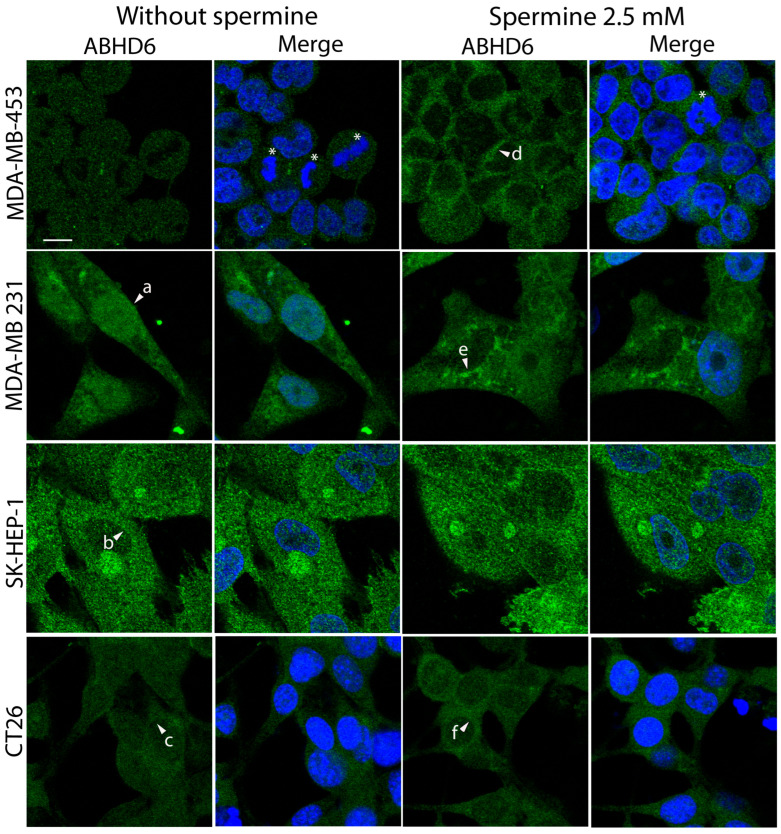
ABHD6 subcellular location varies among different cell lines. Cells were incubated with 2.5 mM spermine at 37 °C for 2 h. Following treatment, cells were fixed, permeabilized, and immunostained for ABHD6 as described in Section 4. The breast cancer cell lines MDA-MB-453 and MDA-MB-231, the liver endothelial cell line SK-HEP-1, and the colon carcinoma cell line CT26 were examined. Arrowheads (a–c) indicate the predominant subcellular localization of ABHD6 under control conditions, as described in the Section 2. Arrowheads (d–f) indicate spermine-induced redistribution toward the perinuclear region. Nuclei were counterstained with DAPI, and asterisks mark mitotic cells identified by condensed chromatin. Images were acquired by confocal fluorescence microscopy using appropriate filter settings for each fluorophore. Scale bar: 10 µm.

Interestingly, immunofluorescence analysis revealed that ABHD6 was predominantly localized in the nucleus in neuroblastoma cells. To further characterize its subcellular distribution, additional cell lines were examined (Figure 2). In the breast cancer cell line MDA-MB-453, ABHD6 was distributed approximately equally between the nucleus and cytoplasm (Figure 2, upper left panel). Similarly to SH-SY5Y cells, a clear nuclear enrichment was also observed in MDA-MB-231 cells (arrowhead a). In contrast, ABHD6 localization was mainly cytoplasmic in the liver endothelial cell line SK-HEP-1 and, to a lesser extent, in the colon carcinoma cell line CT26 (arrowheads b and c, respectively). Spermine treatment induced a redistribution of ABHD6 toward the cytoplasm in breast cancer and colon carcinoma cells, as indicated by increased perinuclear signal and reduced nuclear staining compared with untreated cells (arrowheads d, e and f, respectively). Notably, no detectable ABHD6 translocation was observed in SK-HEP-1 cells upon spermine treatment.

### 2.2. Acid Ceramidase (ACase) Activity Is Modified by ATP13A2-Mediated Spermine Transport

**Figure 3:**
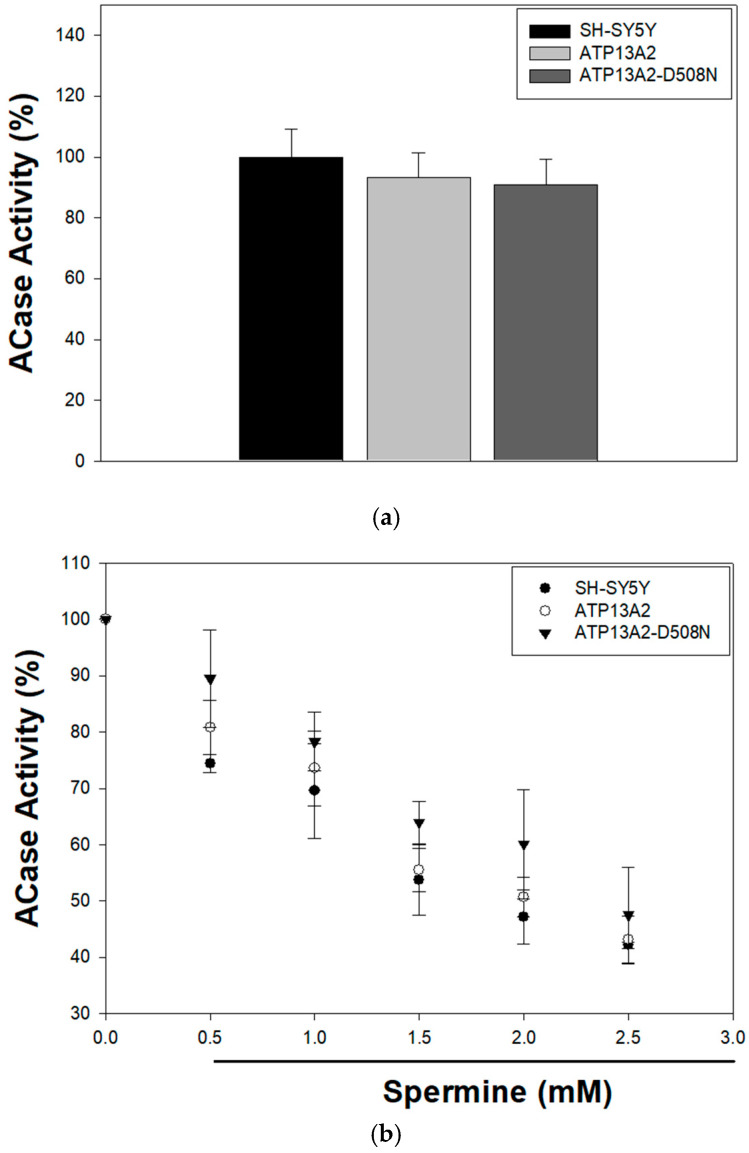

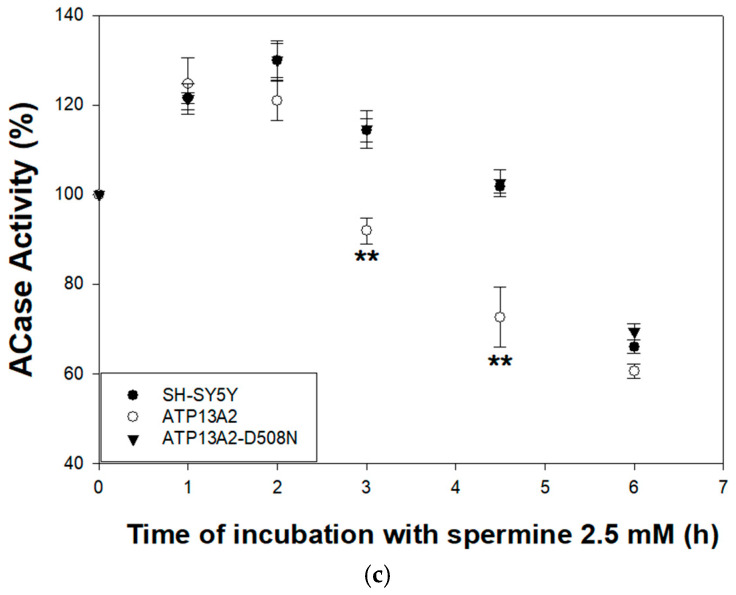
ACase activity is modified by spermine treatment. (**a**) ACase activity was measured in lysosome-enriched fractions as described in Section 4. Data are expressed as a percentage of the activity in SH-SY5Y cells and correspond to the mean ± S.E.M. of six independent experiments performed in duplicate. (**b**) ACase activity was assessed in cells treated with increasing concentrations of spermine (0–2.5 mM) for 24 h at 37 °C. No significant differences between cell lines were detected in panels (**a**–**c**). Cells were incubated with 2.5 mM spermine for the indicated time points. In (**b**,**c**), ACase activity is expressed as percentage relative to untreated cells and represents the mean ± S.E.M. of three independent experiments performed in duplicate. ** *p* < 0.01 vs. SH-SY5Y and ATP13A2-D508N cells at the corresponding time point (one-way ANOVA followed by Newman–Keuls multiple comparisons test).

As described, the degradation of membrane components within ILVs depends on a redistribution process in which cholesterol is exported from the vesicles while BMP levels increase [18,19]. This shift enables specific acid hydrolases—such as ACase, GCase, and acid sphingomyelinase—which carry a positive charge at the acidic pH (~5) of these organelles to interact with the negatively charged ILVs due to the presence of BMP [20]. Since we previously reported that ATP13A2 expression reduces BMP levels [25], and observed here that ABHD6—the enzyme responsible for BMP degradation—is increased in ATP13A2-expressing cells with its subcellular localization altered by spermine (a natural substrate of ATP13A2), we investigated whether spermine treatment influences ACase activity, the acid hydrolase responsible for ceramide catabolism within MVBs and lysosomes. ACase activity was not modified by ATP13A2 expression under basal conditions (Figure 3a). In addition, spermine treatment (0–2.5 mM) decreased ACase activity in a concentration-dependent manner and no significant differences were observed between control SH-SY5Y cells and those expressing either wild-type ATP13A2 or its inactive mutant (Figure 3b). Spermine induced a biphasic response on ACase activity, with an initial increase during the first 2.5 h of incubation followed by a subsequent decline in ATP13A2-expressing cells. A similar biphasic trend was also observed in control SH-SY5Y and ATP13A2-D508N cells, although the reduction in activity occurred only after 4 h of treatment (Figure 3c).

### 2.3. Glucosilcerebrosidase (GCase) Activity Is Increased by ATP13A2 Expression, and Spermine Treatment Decreases Its Activity

**Figure 4:**
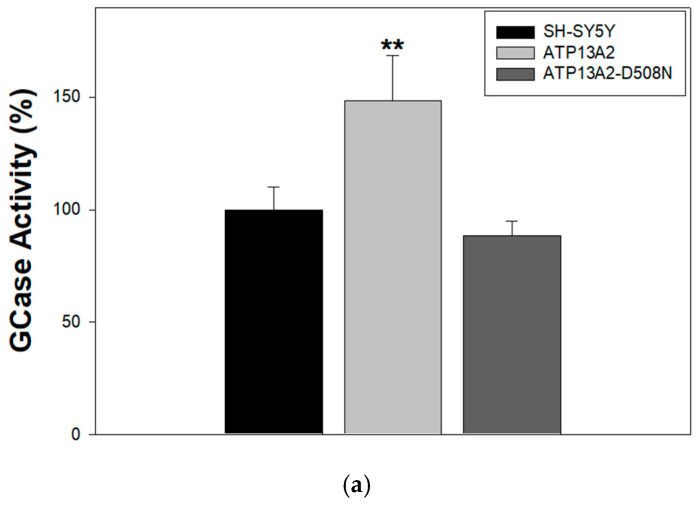

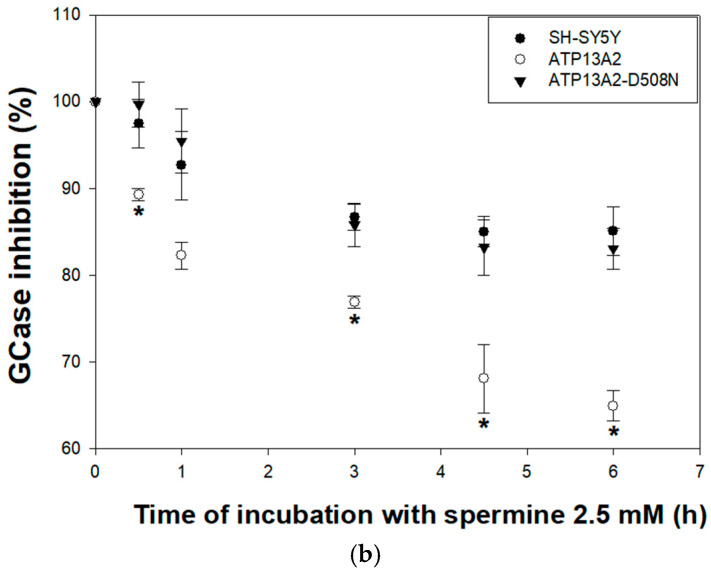
GCase activity is elevated in ATP13A2-expressing cells, and spermine treatment reduces it. (**a**) GCase activity was measured in lysosome-enriched fractions as described in Section 4. Data are expressed as percentage of the activity in SH-SY5Y cells and represent the mean ± S.E.M. of seven independent experiments performed in duplicate. ** *p* < 0.01 vs. SH-SY5Y and ATP13A2-D508N cells; no significant differences were detected between SH-SY5Y and ATP13A2-D508N cells (one-way ANOVA followed by Newman–Keuls multiple comparisons test). (**b**) GCase activity was assessed in living cells incubated with 2.5 mM spermine for the indicated times. Data are expressed as a percentage relative to untreated control cells at the corresponding time point and represent the mean ± S.E.M. of three independent experiments performed in duplicate. * *p* < 0.05 vs. SH-SY5Y and ATP13A2-D508N cells at the corresponding time point (one-way ANOVA followed by Newman–Keuls multiple comparisons test).

To further evaluate how spermine affects endolysosomal lipid digestion, we examined its impact on GCase enzymatic activity. As shown in Figure 4a, ATP13A2 expression resulted in a significant increase in GCase activity compared with SH-SY5Y control cells, indicating a positive association between ATP13A2 and lysosomal lipid degradation. Moreover, GCase activity was significantly higher in ATP13A2 than in ATP13A2-D508N cells, suggesting that this effect depends on the catalytic activity of the transporter. However, spermine addition led to a reduction in GCase activity in all cell types, with a more pronounced inhibitory effect observed in ATP13A2-expressing cells (Figure 4b).

## 3. Discussion

Although ABHD6 is responsible for the degradation of endolysosomal BMP, its catalytic activity is optimal at the neutral pH of the cytosol and does not function as an acid hydrolase [21]. This suggests that it degrades BMP molecules once they have been exported from the ILVs to the cytosolic side of the endolysosomal membrane. It is proposed that this enzyme can attach to charged membrane surfaces through a particularly positively charged region on its structure, this idea would explain its preference for the negatively charged BMP [27]. Here, we observed that ABHD6 levels are increased in ATP13A2-expressing cells and that it is predominantly localized in the nucleus under basal conditions, whereas spermine treatment promotes its redistribution toward the cytoplasm. This is consistent with the decreased BMP levels previously observed in these cells [25] and supports the idea that ATP13A2-mediated spermine export may influence lipid metabolism in the endolysosomal system, considering the crucial role of BMP in this process.

The proposed transport mechanism of ATP13A2 suggests that polyamines might exit the protein through a route embedded in the cytosolic side of the membrane. In this model, the molecule would slide along the protein–lipid interface and reach the cytosol through an opening [11]. A locally thinner membrane region could facilitate the solvation of polyamine positive charges during release [11,13]. Moreover, polyanionic lipids recruited by strongly positively charged surface in the N-terminal domain and other regions of ATP13A2 may assist in detaching the transported molecule from the protein. Such a mechanism could make export more efficient and directional, as lipids forming a seal at the exit site would block the polyamine from re-entering [10,11]. Thus, ATP13A2-mediated polyamine transport may contribute to the formation of platforms enriched in negatively charged lipids, such as BMP, further facilitating spermine export. In turn, BMP redistribution to the cytosolic leaflet would render it accessible to ABHD6-mediated degradation [21] and promote the formation of microdomains required for exosome biogenesis [22].

Palmitoylation and depalmitoylation are reversible post-translational modifications that dynamically regulate protein localization, function, and stability. Palmitoylation often increases protein membrane association and clustering, while depalmitoylation can trigger protein release from membranes or alter protein conformation [28]. These processes play crucial roles in various cellular functions but are particularly important in protein trafficking. Bioinformatic analysis predicts putative palmitoylation/depalmitoylation sites in ABHD6 [29], and its interaction with carnitine palmitoyltransferase 1c (CPT1c) reduces ABHD6 activity and alters its localization [29]. We observed distinct ABHD6 distribution patterns among the cell lines analyzed, indicating that its localization is cell-type dependent under basal conditions. Upon spermine treatment, ABHD6 redistributed toward the cytoplasm in all cell lines except SK-HEP-1 endothelial cells. These findings suggest that the interplay between ABHD6 and spermine leads to divergent outcomes depending on the cellular context and reveal an unexpected behavior that warrants further investigation. Although ABHD6 cytoplasmic redistribution was quantitatively validated, the absence of organelle-specific markers prevents identifying its destination compartment. Future studies should address this limitation, as palmitoylation patterns can differ between cancer cell types [28,30], and polyamines may modulate these modifications indirectly through effects on membrane biophysics.

Although the use of the catalytically inactive ATP13A2-D508N mutant allowed us to differentiate ATP13A2 activity-dependent from activity-independent effects, the absence of ATP13A2 knockout and ABHD6 inhibition models represents a limitation of this study. Therefore, our conclusions support an association between ATP13A2, spermine export, and ABHD6 function, but do not yet establish a definitive causal relationship. Future studies including ATP13A2 gene deletion and ABHD6 loss-of-function approaches are required to validate and refine the mechanistic hierarchy underlying this interplay.

Continuous degradation of membrane components within ILVs requires cholesterol export accompanied by increased BMP levels, enabling positively charged acid hydrolases—such as ACase, GCase, and acid sphingomyelinase—to associate with ILVs [20]. Once bound, these enzymes are protected from proteolysis, ensuring efficient substrate processing. Polycationic compounds including certain antibiotics and antidepressants can disrupt this process by promoting hydrolase dissociation and degradation [31]. Consistent with this, we found that ATP13A2 expression increased GCase activity, aligning with previous observations of decreased GCase activity in ATP13A2-silenced cells [23]. Given that polyamine function is closely linked to their polycationic nature [32], ATP13A2-mediated spermine export may prevent hydrolase dissociation, thereby sustaining lysosomal function. Simultaneous addition of GCase substrate and spermine inhibited enzyme activity in intact cells, likely due to dissociation and degradation of GCase molecules under these conditions. This phenomenon was pronounced in ATP13A2-expressing cells able to uptake more spermine [9,33]. These findings suggest that spermine modulates GCase activity indirectly by altering the lysosomal microenvironment.

In agreement with this model, ACase activity was not affected by ATP13A2 expression but showed dose-dependent inhibition by spermine after 24 h. Surprisingly, a time-course assay revealed a biphasic response in which spermine stimulated ACase activity during the first 2.5 h of incubation in ATP13A2-expressing cells before declining, whereas in control SH-SY5Y and ATP13A2-D508N cells the same profile was observed but with a delayed inhibitory response. The inhibitory effect of spermine on ACase activity can be rationalized in a context where polyamines promote the detachment and degradation of acid hydrolases, leading to a concomitant reduction in enzyme levels, as previously observed for GCase [23]. Notably, increased ATP13A2 expression has been reported in ACase deficient mice [34]. This finding suggests that ATP13A2-mediated polyamine export may help counteract the detrimental accumulation of ceramide resulting from lysosomal ACase deficiency. This is consistent with our previous results showing reduced ceramide levels in ATP13A2-expressing cells [25]. A preliminary report also proposes that ATP13A2 may function as a sphingomyelin flippase [17]. If confirmed, increased ATP13A2 activity would reduce sphingomyelin availability for acid sphingomyelinase, limiting ceramide generation within ILVs, where sphingomyelin is a major substrate for ceramide production [35]. Altogether, these findings indicate that ATP13A2-mediated spermine export can modulate lipid catabolism in the endolysosomal system, reinforcing the concept of a functional interplay between polyamine and lipid metabolism within these organelles.

## 4. Materials and Methods

### 4.1. Materials

Spermine (1141, Sigma-Aldrich, St. Louis, MO, USA); monoclonal anti-ABHD6 antibody (SAB4502488, Sigma-Aldrich); Alexa Fluor 488-conjugated anti-rabbit or anti-mouse IgG secondary antibodies (Thermo Fisher Scientific, Waltham, MA, USA); 4-methylumbelliferyl-β-D-glucopyranoside (M3633, Sigma-Aldrich); p-nitrophenol-N-acetyl-β-D-glucosaminide (N9376, Sigma-Aldrich, St. Louis, MO, USA); RBM14C12 fluorogenic substrate (Avanti Polar Lipids, Alabaster, AL, USA); taurocholic acid (T9034, Sigma-Aldrich). Cell culture reagents were from Sigma-Aldrich unless otherwise specified.

### 4.2. Cell Culture

In this study, the following cell lines obtained from the American Type Culture Collection, Manassas, VA, USA (ATCC) were used: human neuroblastoma SH-SY5Y (CRL-2266), human breast cancer MDA-MB-453 (HTB-131) and MDA-MB-231 (HTB-26), human endothelial SK-HEP-1 (HTB-52), and mouse colon carcinoma CT26 (CRL-2638). SH-SY5Y cell variants transfected by lipofection with the expression vector pcDNA3.1 (SH-SY5Y), stably expressing the human ATP13A2 (ATP13A2) or a mutant of this protein in which the catalytic residue Asp 508 was substituted by Asn (ATP13A2-D508N) were previously obtained [25]. Cells were maintained in Dulbecco’s modified Eagle medium supplemented with 300 mg/mL geneticin (G418) because of the selectable marker provided by the vector, 100 units/mL penicillin, 100 μg/mL streptomycin, and 10% fetal bovine serum (FBS). The cells were grown in humidified 5% CO_2_/air at 37 °C on standard plastic culture dishes.

### 4.3. ABHD6 Detection by Immunofluorescence

Cells (8 × 10^5^) were seeded on glass coverslips and incubated for 24–48 h. Following spermine treatment, cells were washed twice with Phosphate Buffered Saline (PBS) and fixed with 1% PFA in 1 mL of DMEM without FBS for 15 min at 4 °C. A subsequent fixation was performed by adding 1 mL of 4% PFA for 15 min at 4 °C; this step was repeated once. Permeabilization was achieved using 0.1% Triton X-100 in PBS for 5 min at room temperature; the step was repeated once. ABHD6 was detected with a rabbit monoclonal anti-ABHD6 antibody (SAB4502488, Sigma-Aldrich) diluted 1:1000 in 0.1% Triton X-100 in PBS containing 1% bovine serum albumin (BSA), by overnight incubation at 4 °C. After three washes with 0.1% Triton X-100 in PBS, incubation with the Alexa Fluor 488-conjugated goat anti-rabbit IgG secondary antibody (Thermo Fisher Scientific, Waltham, MA, USA) was carried out for 1 h at room temperature. Nuclear counterstaining was performed using 2.5 µM DAPI. Finally, coverslips were washed in PBS and mounted with Fluoromount-G™ (SouthernBiotech, Birmingham, AL, USA), and images were acquired by confocal microscopy (FluoView 1000, Olympus, Tokyo, Japan) using appropriate fluorescence filter settings.

### 4.4. Subcellular Fractionation

Cells (2 × 10^6^ cells/well) were plated in 100 × 20 mm culture dishes and grown to confluence. The medium was then removed, cells were washed once with PBS, and collected by scraping in PBS containing 1 mM etilendiamintetracetic acid (EDTA). The suspension was centrifuged at 1000× *g* for 15 min at 4 °C, and the resulting pellet was homogenized in 3 mL of an isotonic buffer consisting of 10 mM Tris-HCl (pH 7.4), 0.15 M KCl, 1 mM MgCl_2_, 0.25 M sucrose, and a protease inhibitor cocktail (0.1 mM PMSF, 8 µg/mL aprotinin, 1 µg/mL leupeptin). An aliquot of this lysate was used for ABHD6 detection by immunoblotting as described previously [25]. The remaining homogenate was centrifuged at 3000× *g* for 10 min at 4 °C. The supernatant was transferred to a new tube and centrifuged at 25,000× *g* for 30 min at 4 °C to obtain the mitochondrial/microsomal pellet (endolysosome-enriched fraction). This pellet was resuspended in 0.25 M sucrose, 10 mM Tris-HCl (pH 7.4), 0.15 M KCl supplemented with protease inhibitors, homogenized using a Teflon Potter–Elvehjem homogenizer (Wheaton, IL, USA) and aliquots were stored at −80 °C until use. Protein concentration was determined using the Bio-Rad (Hercules, CA, USA) protein assay, with bovine serum albumin as the standard.

### 4.5. Acid Ceramidase Activity Assay

For ACase activity determination in the endolysosome-containing fraction, 75 µL of reaction buffer (100 mM sodium acetate, pH 4.5, containing 20 µM RBM14C12; Avanti Polar Lipids, Alabaster, AL, USA) was mixed with 25 µL of the isolated fraction (20 µg total protein) and incubated for 3 h at 37 °C in opaque 96-well plates (Thermo Fisher Scientific, Waltham, MA, USA). The reaction was stopped by the sequential addition of 50 µL methanol and 100 µL NaIO_4_ (1.25 mg/mL in 200 mM glycine/NaOH buffer, pH 10.6). After 2 h incubation at room temperature in the dark, fluorescence was measured at λ_exc 360 nm/λ_em 446 nm in a microplate multimode reader (Synergy HT, BioTek Instruments Inc., Winooski, VT, USA). To evaluate ACase activity in intact cells, 2.5 × 10^4^ cells/well were seeded in black 96-well plates. After 48 h, cells were incubated with increasing concentrations of spermine (0–2.5 mM) in serum-free medium. The next day, the medium was replaced with 100 µL of fresh serum-free medium containing 30 µM RBM14C12, and cells were incubated for 3 h at 37 °C in a 5% CO_2_ atmosphere. Reactions were stopped with 50 µL methanol followed by 100 µL NaIO_4_, and fluorescence was quantified as described above. For time-course experiments, cells were seeded as described and, after 48 h, incubated with 30 µM RBM14C12 in serum-free medium with or without 2.5 mM spermine. ACase activity was monitored at the indicated time points using the same procedure.

### 4.6. Glucocerebrosidase Activity Assay

GCase activity in the endolysosome-containing fraction was assessed using 20 µg of total protein in 50 µL of reaction buffer containing 0.25% Triton X-100 (*v*/*v*), 0.25% taurocholic acid (*w*/*v*), 1 mM EDTA, 1% BSA, and 1 mM 4-methylumbelliferyl β-D-glucopyranoside (M3633, Sigma-Aldrich) in citrate/phosphate buffer (pH 5.4). After 40 min at 37 °C, the reaction was stopped with an equal volume of 1 M glycine (pH 12.5). An aliquot of 100 µL was transferred to black 96-well plates, and fluorescence was recorded at λ_exc 355 nm/λ_em 460 nm using a multimode microplate reader (Synergy HT, BioTek Instruments Inc., Winooski, VT, USA). For GCase activity measurements in intact cells, 2.5 × 10^5^ cells/well were plated in black 96-well plates. After 48 h, cells were incubated at 37 °C for 0–6 h in 50 µL of reaction buffer containing 1 mM 4-methylumbelliferyl β-D-glucopyranoside prepared in equal volumes of PBS and 100 mM sodium acetate buffer (pH 4.5), with 2.5 mM spermine added when indicated. Reactions were stopped by adding 50 µL 1 M glycine (pH 12.5), and liberated 4-methylumbelliferone was quantified as described above.

### 4.7. Statistical Analysis

Statistical analyses were performed using GraphPad Prism 6 (GraphPad Software, San Diego, CA, USA). The number of biological replicates and independent experiments for each data set is indicated in the corresponding figure legends. Comparisons among experimental groups were made using one-way ANOVA followed by the Newman–Keuls multiple comparisons test. Data are expressed as mean ± SEM, and a *p* < 0.05 was considered statistically significant.

## 5. Conclusions

In summary, ATP13A2-mediated spermine export appears to influence both ABHD6 localization and the activity of lysosomal hydrolases, suggesting that ATP13A2 contributes to the functional connection between polyamine homeostasis and lipid metabolism within the endolysosomal system. These findings reveal an underappreciated interplay between two key metabolic pathways that may have important implications for lysosomal function in both physiological and pathological contexts.

## Data Availability

The data that support the findings of this study are available in CONICET’s Institutional Digital Repository https://ri.conicet.gov.ar/handle/11336/271756, (accessed on 10 December 2025).

## Supplementary Materials

The following supporting information can be downloaded at https://www.mdpi.com/article/10.3390/ijms27010484/s1.

## Abbreviations

The following abbreviations are used in this manuscript:

SH-SY5Y	Human neuroblastoma cell line
PBS	Phosphate-Buffered Saline
BMP	bis(monoacylglycero)phosphate
EDTA	etilendiamintetracetic acid
BSA	bovine serum albumin
FBS	fetal bovine serum
