# Supplementary figures and images for: ATP13A2-Mediated Spermine Export Modulates Lipid Catabolism in the Endolysosomal System of SH-SY5Y Cells
Source: Int J Mol Sci. 2026 Jan 2;27(1):484. doi: 10.3390/ijms27010484 (PMC12787256; doi:10.3390/ijms27010484)

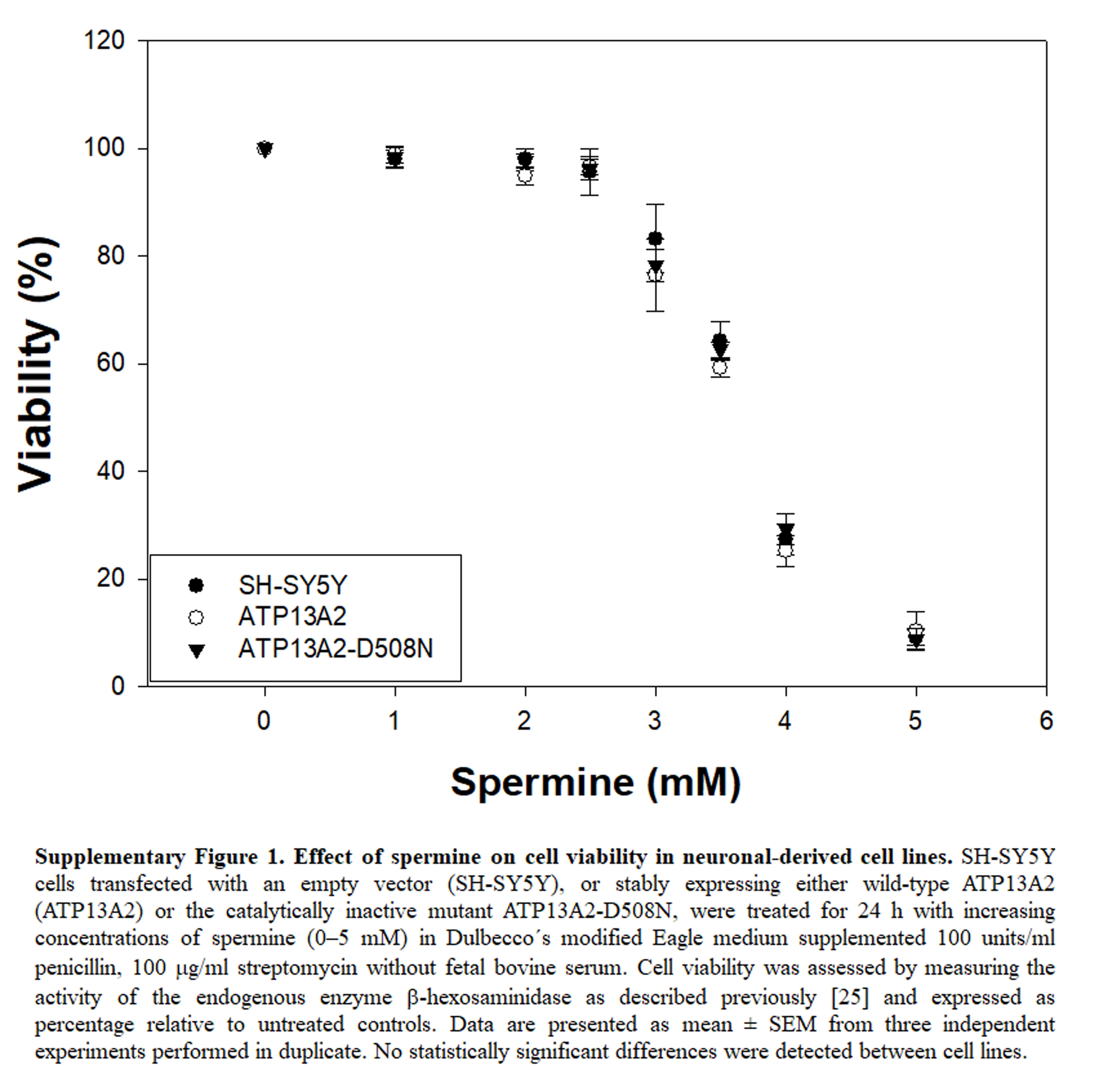

Supplement: Supplementary file 1 [file ijms-27-00484-s001.zip › ijms-3924306-supplementary.tif]
